# Genomic and Phenotypic Characteristics in Geographically Separated Clinical *Campylobacter jejuni* ST353CC Isolates

**DOI:** 10.3390/microorganisms9122540

**Published:** 2021-12-08

**Authors:** Cecilia Johansson, Christian Kampmann, Anna Nilsson, Johan Dicksved, Lars Engstrand, Hilpi Rautelin

**Affiliations:** 1 Clinical Microbiology, Department of Medical Sciences, Uppsala University, SE-75185 Uppsala, Sweden; cecilia.johansson@medsci.uu.se (C.J.); christian.kampmann@medsci.uu.se (C.K.); anna.nilsson@slu.se (A.N.); 2 Department of Animal Nutrition and Management, Swedish University of Agricultural Sciences, SE-75007 Uppsala, Sweden; johan.dicksved@slu.se; 3 Centre for Translational Microbiome Research, Department of Microbiology, Tumor and Cell Biology, Karolinska Institute, SE-17177 Stockholm, Sweden; lars.engstrand@ki.se

**Keywords:** *Campylobacter jejuni*, ST353CC, virulence, pathogenesis, microbiota, whole-genome sequence

## Abstract

*Campylobacter jejuni* fecal isolates of eight international travelers, 5 of which had traveled to Ecuador and 3 to Bangladesh, were characterized, and the possible relationship between bacterial traits and clinical symptoms was further analyzed. All eight isolates belonged to the same Multi-Locus Sequence Type clonal complex (ST353CC). The three isolates from Bangladesh were all of the same sequence type (ST-9438), and when compared to isolates of various other sequence types, they had a larger quantity of unique genetic content, higher expression levels of some putative virulence genes involved in adhesion and invasion (*flp*A, *cia*B and *iam*A), and showed higher adhesion levels to human HT-29 colon cancer cells in an in vitro infection model. However, in contrast to the seemingly higher pathogenic potential of these bacterial isolates, travelers infected with the ST-9438 isolates had no or only very mild symptoms, whereas the other individuals, whose bacterial isolates seemed to have less pathogenic potential, generally reported severe symptoms. When studying the 16S rRNA gene-based fecal microbiota in samples collected prior to travel, there was an individual variation in the relative abundance of the three major bacterial phyla Actinobacteria, Bacteroidetes and Firmicutes, but there were no associations between composition and diversity of microbiota and development of severe symptoms from the infection. It remains to be confirmed by larger studies whether an individual’s characteristics such as gut microbiota, might be related to the severity of symptoms in *Campylobacter* infections.

## 1. Introduction

*Campylobacter* is the most common cause of acute human bacterial gastroenteritis globally, and since 2005 it has been the most reported zoonosis in the European Union, with over 246,000 cases in 2018 [1]. It has been estimated that approximately 1% of European inhabitants develop campylobacteriosis every year [2].

*C. jejuni* is the most common pathogen in the genus *Campylobacter*, and *Campylobacter* infection usually develops a few days after ingestion of the bacteria. The severity of the disease varies, and symptoms include fever, headache, vomiting, and abdominal pain, in addition to watery or sometimes even bloody diarrhea [3]. Post-infectious sequelae, such as reactive arthritis [4], irritable bowel syndrome [5], and the severe autoimmune demyelinating neuropathy Guillain–Barré syndrome [6] may occur, which substantially increases the burden of the disease.

The *C. jejuni* population structure is commonly described using Multi-Locus Sequence Typing (MLST), and to date over 10,000 sequence types (STs) have been identified [7]. STs can be further grouped into clonal complexes (CCs), where some CCs are host- or niche-specific, i.e., specialists, and others are considered generalists that can colonize a wide variety of hosts worldwide [8]. The generalists ST21CC and ST45CC are the most common CCs among clinical isolates [9,10].

The mechanisms of *Campylobacter*–host interaction and pathogenesis are largely unclear. Putative virulence factors involved in, for example, capsule biosynthesis, motility and chemotaxis, adhesion and invasion, and stress responses have been proposed as contributors to the infection. The processes of adhesion and invasion have been well-studied. The bacterial adhesins CadF and FlpA bind fibronectin on the host-cell surface to initiate adhesion [11,12,13,14], while invasion factors such as CiaB and IamA are considered to be involved in the later stages of invasion [15,16,17]. The large number of *Campylobacter* whole-genome sequences available in public repositories has facilitated screening for the presence of putative virulence factors in individual isolates. Despite this, there is still no clear correlation between the mere presence of a gene, the virulence traits of that particular isolate, and the severity of infection. Instead, some studies on the gene expression of putative virulence genes have shown a correlation between the expression levels of genes involved in adhesion and invasion, and their level of adhesion to human cells in vitro [18,19].

To further characterize pathogenesis mechanisms, studies on bacteria themselves can be combined with in vitro infection studies using human cell lines. Adhesion and invasion of a particular *Campylobacter* isolate, and resultant downstream cellular processes such as the induction and release of specific cytokines, can be monitored. We have previously used HT29 cells, and a human colon cancer epithelial cell line for such studies, and have shown differences in adhesion-ability between different isolates of both *C. jejuni* [20,21] and *C. coli* [19,22].

In addition to the characteristics of enteropathogens, aspects of the host such as the composition of intestinal microbiota, may be crucial for the development of intestinal infections. We have previously shown differences between the composition of fecal microbiota from individuals who later became infected with *Campylobacter*, and those who remained uninfected [23,24].

In our previous study, we described *C. jejuni* infection in 14 international travelers [24]. Here, we show that eight of these individuals, although infected in two remote destinations (Bangladesh and Ecuador) harbored *C. jejuni* isolates belonging to the same MLST ST353CC. Our aim was to study various bacterial characteristics, and to analyze the possibility of bacterial traits being associated with symptoms in the infected travelers.

## 2. Materials and Methods

### 2.1. Data on Participants and Microbiota Analyses

This investigation was part of a larger study that is described above [24]. Data on eleven individuals infected with *Campylobacter* (Table 1) were extracted from questionnaires collected prior to this investigation. None of the participants had taken antibiotics within three months before taking a fecal sampling for the microbiota analyses. Symptoms as described by the participants in the questionnaires, were scored by a specialist in infectious diseases (CK), in compliance with existing guidelines [25,26], on a scale of one to five where 1 stood for no symptoms and 5 stood for the most severe symptoms (Table 2). The travelers to Ecuador lived together for several weeks before traveling and ate communal meals.

Written informed consent was obtained from each individual. The regional board of the ethics committee at Uppsala University approved the study (Dnr 2011/254).

The microbiota dataset was retrieved from an already-published study where 16S rRNA gene amplicons were compared between travelers who remained non-infected, and those who became infected during a trip to destinations with a high estimated risk of obtaining an enteric infection [24]. Thus, the procedure used for preparation of the fecal samples for amplification of the V3–V4 region of the 16S rRNA gene, has been described elsewhere [24].

### 2.2. Bacterial Culture Conditions

*Campylobacter* isolates had been isolated prior to this study [24]. Bacteria were grown microaerobically (Oxoid CampyGen, Thermo Fisher Scientific, Waltham, MA, USA) at 42 °C, and routinely resuscitated from frozen stocks by first growing overnight on blood agar (Columbia agar plates supplemented with 5% horse blood: Oxoid, Basingstoke, UK), followed by overnight incubation in Brucella broth (Becton, Dickinson and Company, Franklin Lakes, NJ, USA). To collect bacteria, cultures were centrifuged at 8000× *g* for 5–10 min.

### 2.3. Whole-Genome Sequencing

Total bacterial DNA was extracted using the MagNa Pure Compact Nucleic Acid isolation kit I (Roche, Penzberg, Germany) according to the manufacturer’s protocol (version 12). Of the eleven original isolates, two could not be resuscitated so DNA extracted from *Campylobacter* isolates during a previous study [24] was used. The libraries for whole genome sequencing were prepared with a Nextera XT sample preparation kit (Illumina, San Diego, CA, USA). An Illumina MiSeq platform, with 2 × 300 paired-end reads, was used for whole-genome sequencing. The single-reads were assembled to contigs with Velvet version 7.0.4 [27] running as a plugin in Geneious version 8.1.9 (https://www.geneious.com).

### 2.4. Genomics

New whole-genome sequences from this study were submitted to the Genbank database [28] (BioProject number PRJNA713788), and accession numbers are shown in Table 1. MLST-typing in this study was based on seven house-keeping genes, and performed by a sequence query of the whole-genome sequences against the PubMLST database [7] (last accessed: 5 September 2021). The allelic profiles of four of the isolates (three were identical) did not match any of the existing STs in the PubMLST database, and were submitted to PubMLST for assignment. The whole-genome phylogenetic trees were constructed in SplitsTree [29], using the average nucleotide identity (ANI) to closely related taxa calculated using the Gegenees software version 2.2.1 with a threshold of 20% [30]. Sequence alignments, ORF annotations, translations and phylogenetic analyses of individual genes were performed in the CLC Main Workbench (Qiagen, Hilden, Germany) using standard settings. The BLAST Ring Image Generator (BRIG) application was used to visualize similarities between pTet plasmids [31].

### 2.5. RNA Preparation from Bacteria and cDNA Synthesis

Bacterial RNA was extracted from overnight cultures using the ISOLATE II RNA Mini Kit (Bioline Reagents Ltd., London, UK) according to the manufacturer’s protocol. DNase I treatment (Ambion by life technologies, Carlsbad, CA, USA) was performed both on-column and on the final eluted RNA. The concentration of RNA was determined using Nanodrop, and RNA integrity was verified on a 1% agarose gel. A Maxima First Strand cDNA Synthesis Kit for qPCR (Thermo Fisher Scientific, Waltham, MA, USA) was used to reverse transcribe 1 µg of RNA according to the manufacturer’s protocol. A minus-RT control reaction was set up for each sample to rule out residual DNA. For qPCR 1/2000 of the cDNA synthesis reaction was used. At least two independent RNA preparations were used for real-time qPCR.

### 2.6. Quantitative PCR

Real-time qPCR was performed in the Bio–Rad CFX96 Touch cycler using the DyNAmo HS SYBR Green qPCR kit (Thermo Fisher Scientific, Waltham, MA, USA). Primer sequences for virulence genes are available upon request. DNA and cDNA samples were diluted 10–100 times prior to amplification. For virulence gene expression analyses, the expression levels of each gene were first normalized to that of 16S rRNA for each sample and run. Thereafter, expression levels were expressed relative to that of the *C. jejuni* 11168 reference strain. For each isolate, four biological replicates were available and one to two qPCRs were run for each replicate resulting in a total of six qPCRs per isolate. Results are presented as the mean for all replicates.

### 2.7. Cell Culture Conditions

The HT-29 human colon cancer cell line (ECACC 91072201) was maintained in RPMI 1640 media (Gibco by life technologies, Carlsbad, CA, USA) and supplemented with 2 mM glutamine (Swedish National Veterinary Institute, Uppsala, Sweden), 10% fetal bovine serum (FBS, Gibco by life technologies, Carlsbad, CA, USA), 100 U/mL penicillin, and 100 µg/mL streptomycin (Swedish National Veterinary Institute, Uppsala, Sweden).

### 2.8. In Vitro Cell Adhesion Assay and IL-8 ELISA

Adhesion to HT-29 cells, and IL-8 levels in the media were measured at 3 h post-infection, as previously described [20]. Results are presented as the mean of three infections.

## 3. Results

### 3.1. Phylogenetic Characterization of C. jejuni Isolates

**Figure 1:**
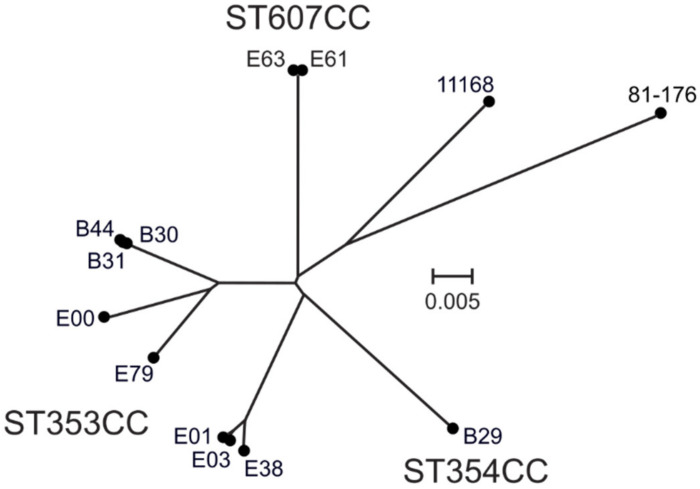
ANI-based whole-genome sequence phylogenetic tree of the eleven isolates sequenced in this study, together with the *C. jejuni* 11168 and 81-176 reference strains, showing the separation of ST353CC, ST354CC, and ST607CC.

Viable *C. jejuni* isolates were available from nine out of the 14 originally infected individuals [24], while from two of the individuals only *Campylobacter* DNA was available (Table 1). All eleven isolates for which DNA was available, were whole-genome sequenced. The CC and ST of each isolate was determined through a sequence query of the whole genome sequence against the PubMLST database [7]. Eight of the isolates, acquired from Bangladesh or Ecuador, were shown to belong to the same CC, ST353CC, while one isolate from Bangladesh belonged to ST354CC, and two isolates from Ecuador belonged to ST607CC (Table 1). Phylogenetic analyses of the whole-genome sequences confirmed the clustering of isolates, and revealed a separation of isolates from Bangladesh and Ecuador within ST353CC (Figure 1).

### 3.2. Genomic Characterization of C. jejuni ST353CC Isolates

Eight isolates belonged to the same clonal complex (ST353CC), of which three isolates from Bangladesh and two isolates from Ecuador even belonged to the same sequence types, ST-9438 and ST-9336, respectively (Table 1). These eight isolates were chosen for further genomic characterization.

Extensive in silico screening confirmed the presence of over 100 previously identified genes suggested to play a role in virulence, and involved in capsule biosynthesis, motility, chemotaxis, adhesion, invasion, stress response, and iron acquisition in all of the isolates (Supplementary Materials Table S1). Interestingly, the E00 isolate seemed to lack both the *flaA* and *flaB* genes that encode flagellin. Further in silico analyses of the putative virulence genes *cadF*, *flpA*, *iamA*, *ciaB*, *ceuE*, and *cdt* revealed a high level of similarity at both nucleotide and amino acid sequence levels in all the isolates, with only a few amino acids differing in each protein. However, five isolates (B30, B31, B44, E00, and E79) had an R135C substitution in the fibronectin-binding site (FRLS) of the CadF protein, due to a single nucleotide substitution (C403T) in the *cadF* gene.

Comparative genomic analysis was performed to identify genome regions that were unique to isolates belonging to different sequence types within ST353CC. Thirty annotated genes unique to the ST-9438 isolates from Bangladesh were detected (Supplementary Materials Table S2). Functional characteristics of each gene were assigned according to the Clusters of Orthologous Groups (COG) database for *C. jejuni* [32]. This revealed that ST-9438 isolates had a larger fraction of unique genes involved in information storage and processing than the isolates belonging to other STs.

The largest region unique to the ST-9438 isolates (B30, B31, and B44) consisted of approximately 50 k base pairs. BLASTn analyses showed that this region contained *virB4*, *virB5*, *virB6*, *virB8*, *virB10*, *virB11*, and *virD4* genes, and was almost identical to the pTet plasmid detected in the *C. jejuni* reference strain 81-176, which is known to be highly invasive, and harbored *tetO* and *topIII* genes, a helicase, and an ATPase (Supplementary Materials Figure S1). Further assemblies, plasmid isolation, and agarose gel electrophoresis confirmed that this region exists as a circularized plasmid of 50.3 k base pairs in the ST-9438 isolates. The *tetO* gene was also found in the non-ST-9438 isolates, but at a different and chromosomally integrated location (Supplementary Materials Figure S2).

### 3.3. Various Symptom Scores in Travelers Infected with C. jejuni ST353CC Isolates

**Figure 2:**
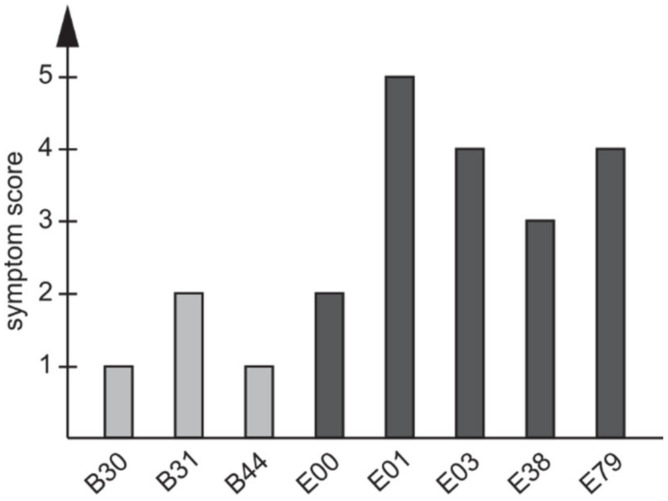
Symptom scores in individuals infected with *C. jejuni* ST353CC isolates. Three individuals (B30, B31, and B44) were infected with ST-9438 isolates and had traveled to Bangladesh. The rest of the individuals had traveled to Ecuador. Symptoms score 1–5: 1 indicates no symptoms, and 5 indicated the most severe symptoms.

As the eight travelers were infected with bacterial isolates belonging to ST353CC, we wanted to see if the severity of symptoms was similar. Therefore, the severity of symptoms was scored. All infected individuals were asked to fill out questionnaires and to describe their symptoms in detail [24]. The reported symptoms were scored on a scale of 1 to 5, where 1 corresponded to no symptoms, and 5 to the most severe symptoms (extensive diarrhea, fever, and reduced general health) (Table 2). The mean symptom score for all of the eight individuals infected with ST353CC isolates was 2.7. However, on the individual level, the symptom scores ranged from 1 to 5 (Figure 2). Three of the eight individuals were all infected with isolates belonging to ST-9438 and had reported no (score 1) or only mild (score 2) symptoms (Figure 2), whereas the remaining five travelers mostly reported more severe symptoms (4 of 5 with a score ≥3, Figure 2).

### 3.4. Pre-Travel Composition of the Fecal Microbiota in Travelers Infected with C. jejuni ST353CC Isolates

To further study the characteristics of travelers later infected with related *C. jejuni* ST353CC isolates, the pre-travel fecal microbiota results [24] of these individuals were compared. The bacterial alpha-diversity, expressed using the Shannon H index, did not differ between the individuals infected with ST-9438 isolates, and those that were not, in fecal samples collected before traveling. Moreover, prior to travel there was no difference in the alpha-diversity of the fecal microbiota for individuals who later reported no, or only minor symptoms (1–2 on the symptom scale) when compared to those who developed severe symptoms (3–5 on the symptom scale).

**Figure 3:**
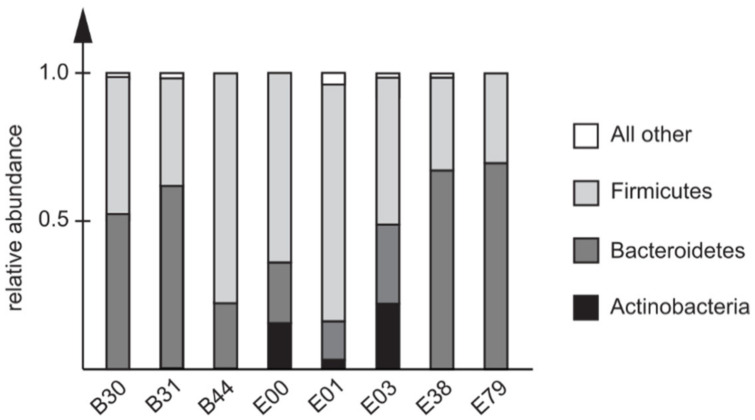
Composition of fecal microbiota prior to travel in individuals later infected with *C. jejuni* ST353CC isolates. The relative abundance of detectable phyla is shown for each individual. Three individuals (B30, B31, and B44) included meat in their diet whereas the rest of the subjects adhered to a non-meat diet.

Three major bacterial phyla were detected in the fecal samples collected prior to traveling: Actinobacteria, Bacteroidetes, and Firmicutes. There was a clear individual variation in the relative abundances but, in line with the alpha-diversity data, there were no apparent correlations between microbial community compositions prior to the travel, and the development of severe symptoms (Figure 3).

### 3.5. In Vitro Characteristics of C. jejuni ST353CC Isolates

Six viable ST353CC isolates, three from individuals infected with ST-9438 isolates who exhibited only minor symptoms (B30, B31, and B44), and three from individuals infected with isolates belonging to various STs who exhibited severe symptoms (E03, E38 and E79), were available for phenotypic analyses (Table 1). These isolates were analyzed for in vitro characteristics together with the *C. jejuni* 11168 and 81-176 reference strains.

**Figure 4:**
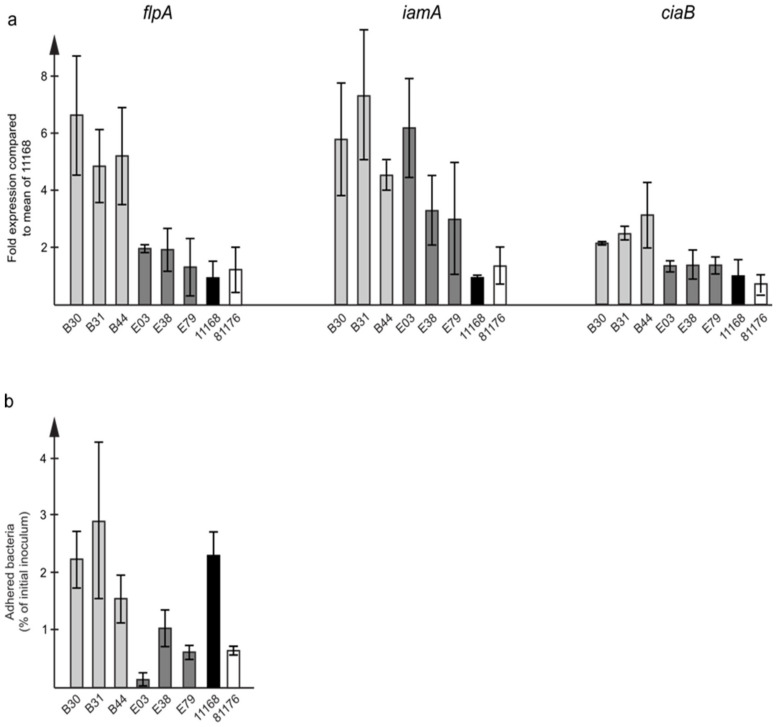
In vitro bacterial characteristics of *C. jejuni* ST353CC isolates. (**a**) RNA expression of putative virulence genes *flp*A, *iam*A, and *cia*B involved in adhesion and invasion, analyzed by RT-qPCR. Data are expressed relative to the *C. jejuni* 11168 reference strain, and shown as mean of six PCR-experiments for each isolate together with data for the 11168 and 81-176 reference strains. (**b**) HT-29 cells were infected with *C. jejuni* ST353CC isolates at an MOI (multiplicity of infection) of 100. The *C. jejuni* 11168 and 81-176 reference strains were included for comparison. Adhered bacteria at 3 h post-infection were quantified using qPCR and expressed as a percentage of the starting inoculum. The graph shows the mean of three independent infection experiments for each isolate.

When the expression of virulence genes involved in adhesion and invasion was measured, ST-9438 isolates had higher expression levels of the *flpA*, *ciaB* and *iamA* genes (Figure 4a), while the expression of *cadF* was similar in both groups.

The adhesion of the isolates to human HT-29 colon cancer cells was assessed using our previously established in vitro infection model [20]. The ST-9438 isolates clearly had higher levels of adhesion compared to the others (Figure 4b). However, the levels of induced IL-8 in the cells were similar for both groups.

## 4. Discussion

*C. jejuni* isolates of eleven healthy young individuals who had travelled to Bangladesh or Ecuador [24], were whole-genome sequenced and shown to belong to three globally distributed CCs: ST353CC, ST354CC, and ST607CC [33,34,35]. Despite the remote travel destinations and the high molecular diversity of the *C. jejuni* population, eight isolates, three from Bangladesh and five from Ecuador, were found to be genetically related, as they belonged to the same clonal complex (ST353CC). Here, we analyzed these ST353CC isolates as well as some characteristics of the infected hosts.

The clonal complex ST353CC accounts for 4.4% of all *C. jejuni* isolates in PubMLST and is globally distributed [7]. In our comparative genomic analysis, we identified differences between the ST353CC isolates. Three isolates, which all belonged to the same sequence type (ST-9438), were associated with less severe symptoms but had higher quantities of unique genetic content compared to the isolates relating to more severe infections. This is in line with our earlier findings on clinical ST21CC isolates, which harbored less accessory genetic content when isolated from blood in severe infections, when compared to isolates cultivated from fecal samples only [21].

As far as putative virulence genes were concerned, most that are involved in capsule biosynthesis, motility, chemotaxis, adhesion, invasion, stress response, and iron acquisition were equally present in all samples. To better understand the role of these genes in virulence, we looked at the expression levels of selected genes involved in adhesion and invasion. Higher expression levels for these genes were detected in the ST-9438 isolates despite the lower symptom scores in travelers who were infected with these isolates. Furthermore, in our in vitro infection model, higher levels of attached bacteria were demonstrated for the ST-9438 isolates. These findings, together with the results from our previous study on *C. coli* [19], show a direct correlation between the expression levels of genes relating to adhesion, and actual adherence capacity to human cells in an in vitro assay.

For *cadF*, which codes for the fibronectin-binding protein involved in adhesion, the expression levels were similar for all ST353CC isolates, irrespective of sequence type. However, we detected an interesting difference in the amino acid sequence of the CadF protein. The ST-9438 isolates had an R135C substitution in the fibronectin-binding site FRLS [36], possibly altering the fibronectin-binding capacity and contributing to their higher adhesion levels.

Altogether, our results emphasize the importance of looking at gene expression levels and gene product functions, rather than merely demonstrating the presence of a gene to understand the impact of experimental results regarding virulence. However, the picture as a whole seems to be even more complicated, and far from clear, as an inverse correlation was demonstrated between the levels of bacterial adhesion to human cells in vitro, and the severity of symptoms in individuals infected with the corresponding isolate.

Bacterial pathogenesis in general, and *Campylobacter* infection in particular, is difficult to study as the development of infection is not only influenced by bacterial characteristics, but is very likely to be influenced by characteristics of the host as well, including factors such as the composition of intestinal microbiota. Studies using murine infection models have indicated a correlation between the composition of intestinal microbiota and *Campylobacter* infection [37]. We have previously shown that the composition of intestinal microbiota had a significant impact on the susceptibility of humans to *Campylobacter* infection [23,24]. For instance, the relative abundance of *Bacteroides* was significantly higher among poultry abattoir workers who later became *Campylobacter*–positive as compared to subjects who remained uninfected [23].

In the present study, there were no apparent differences in either microbial diversity or community composition for study participants who later developed severe symptoms, compared to those with minor symptoms. Although no consistent difference has been found in the overall bacterial composition of the microbiota of vegetarians and vegans compared to omnivores, an increased abundance of Bacteroidetes, and of OTUs belonging to this phylum, has been demonstrated in vegetarians and vegans elsewhere [38,39,40,41].

It remains to be studied in a much larger number of individuals whether diet, or if specific microbial taxa, could be related to the development of more severe symptoms in *Campylobacter*–infected subjects, or whether *C. jejuni* isolates of ST-9348 cause less severe symptoms. After all, the comparative genomic analyses did not identify any specific or unique genes in the isolates that could have explained the more severe symptoms in infected individuals.

## 5. Conclusions

Here, we analyzed the bacterial and host characteristics for a very limited number of international travelers infected with genetically related (ST353CC) *C. jejuni* isolates. Travelers infected with ST-9438 isolates developed no, or only mild, symptoms. This is despite their *Campylobacter* isolates seeming to have more pathogenic potential in vitro compared to isolates of other sequence types within ST353CC, which had infected travelers who developed severe symptoms. Whether an individual’s characteristics (such as gut microbiota) might be related to the severity of symptoms in *Campylobacter* infection needs to be confirmed by much larger studies.

## Figures and Tables

**Table 1:** *Campylobacter*–positive individuals and their *C. jejuni* isolates (*n* = 11).

Travel Destination	Gender	Age (Years)	Isolate	CC/ST ^#^	Accession
Bangladesh	F	24	B29	354/354	JAGFPS000000000
	F	24	B30	353/9438 ^	JAGFPT000000000
	F	28	B31	353/9438 ^	JAGFPR000000000
	F	26	B44	353/9438 ^	JAGFPU000000000
Ecuador	F	29	E00 *	353/462	JAGFPV000000000
	M	24	E01 *	353/9336	JAGFPN000000000
	F	20	E03	353/9336	JAGFPO000000000
	F	20	E38	353/9437 ^	JAGFPP000000000
	F	23	E61	607/607	JAGFPQ000000000
	F	19	E63	607/607	JAGFPL000000000
	F	22	E79	353/3515	JAGFPM000000000

(^#^ according to MLST assignment; * only DNA available; ^ novel STs).

**Table 2:** Scoring of intestinal symptoms based on questionnaires filled out by travelers.

Score	Symptom	Description
1	No symptoms	
2	Mild symptoms	Slight abdominal discomfort, ≤3 loose stools in 24 h, symptoms did not force any change in planned daily activities
3	Severe diarrhea	>3 loose stools in 24 h + enteric symptoms (e.g., fecal urgency, nausea, abdominal pain/cramps, emesis), symptoms did not force a significant change in planned daily activities, duration ≤3 consecutive days
4	Severe diarrhea	>3 loose stools in 24 h + enteric symptoms for >3 consecutive days, symptoms forced a significant change in planned daily activities and/or affected general health
5	Severe diarrhea	Bloody diarrhea, and/or fever, and/or severely affected general health

If no exact fecal frequency was provided, score was based on the total information and graded down.

## Data Availability

All whole-genome sequences are available in GenBank [28] under the accession numbers shown in Table 1. Primers are available from the authors upon request. Additional data supporting the conclusions of this article are included within the article (and its Supplementary Materials).

## Supplementary Materials

The following are available online at https://www.mdpi.com/article/10.3390/microorganisms9122540/s1, Figure S1: BRIG analysis of plasmids identified in *C. jejuni* ST353CC isolates B30, B31 and B44 compared to the pTet plasmid from *C. jejuni* 81-176, Figure S2: Chromosomal location of a 2862 nucleotide long fragment containing the tetO gene in non-ST-9438 isolates, Table S1: Screening of putative virulence genes in *C. jejuni* ST353CC isolates sequenced in this study, Table S2: Results from comparative genomic analyses identifying genes unique for the *C. jejuni* ST353CC isolates sequenced in this study.
