# Supplementary material for: Genomic and Phenotypic Characteristics in Geographically Separated Clinical Campylobacter jejuni ST353CC Isolates
Source: Microorganisms. 2021 Dec 8;9(12):2540. doi: 10.3390/microorganisms9122540 (PMC8709058; doi:10.3390/microorganisms9122540)

Supplementary material

Table S1. Screening of putative virulence genes in *C. jejuni* ST353CC isolates sequenced in this study. Compared to *C. jejuni* reference strains 11168 (GenBank AL111168.1), 81-176 (GenBank *CP000538.1 (chromosome) and **AY394561.1 (pTet plasmid), and 108 (Genbank #JX436460.1).

**Function**	**Gene annotation in 11168, 81-176*/** or 108#**		**Analysis of 353CC isolates**		
	**Locus tag**	**Gene name**	**Present in all isolates**	**Not found in any isolates**	**Differences**
Capsule biosynthesis	cj1413c	*kpsS*, capsule polysaccharide modification protein	✓		
	cj1414c	*kpsC*, capsule polysaccharide modification protein	✓		
	cj1415c	*cysC*, putative adenylylsulfate kinase	✓		
	cj1423c	*hddC1*, putative D-glycero-D-manno-heptose 1-phosphate guanosyltransferase			Present in B30, B31 and B44
	cj1424c	*gmhA2*, phosphoheptose isomerase		✓	
	cj1425c	*hddA*, putative D-glycero-D-manno-heptose 7-phosphate kinase			Present in E00, E79, B30, B31 and B44
	cj1428c	*fcl*, GDP-L-fucose synthetase		✓	
	cj1430c	*rfbC*, putative dTDP-4-dehydrorhamnose 3,5-epimerase			Present in B30, B31 and B44
	cj1431c	*hddC2*, capsular polysaccharide heptosyltransferase		✓	
	cj1439c	*glf*, UDP-galactopyranose mutase		✓	
	cj1441c	*kfiD*, UDP-glucose 6-dehydrogenase		✓	
	cj1443c	*kpsF*, D-arabinose 5-phosphate isomerase	✓		
	cj1444c	*kpsD*, capsule polysaccharide export system periplasmic protein	✓		
	cj1445c	*kpsE*, capsule polysaccharide export system inner membrane protein	✓		
	cj1447c	*kpsT*, capsule polysaccharide export ATP-binding protein	✓		
	cj1448c	*kpsM*, capsule polysaccharide export system inner membrane protein	✓		
Motility and	cj0041	*fliK*, putative flagellar hook-length control protein	✓		
	cj0042	*flgD*, putative flagellar hook assembly protein	✓		

chemotaxis	cj0043	*flgE*, flagellar hook protein	✓
	cj0059c	*fliY*, putative flagellar motor switch protein	✓
	cj0060c	*fliM*, flagellar motor switch protein	✓
	cj0061c	*fliA*, RNA polymerase sigma factor for flagellar operon	✓
	cj0063c	*flhG*, flagellar biosynthesis protein, ATPase	✓
	cj0064c	*flhF*, flagellar biosynthesis protein	✓
	cj0195	*fliI*, flagellum-specific ATP synthase	✓
	cj0283c	*cheW*, chemotaxis protein	✓
	cj0284c	*cheA*, chemotaxis histidine kinase	✓
	cj0285c	*cheV*, chemotaxis protein	✓
	cj0318	*fliF*, flagellar M-ring protein	✓
	cj0319	*fliG*, flagellar motor switch protein	✓
	cj0320	*fliH*, putative flagellar assembly protein	✓
	cj0335	*flhB*, flagellar biosynthesis protein	✓
	cj0336c	*motB*, putative flagellar motor protein	✓
	cj0337c	*motA*, putative flagellar motor proton channel	✓
	cj0351	*fliN*, flagellar motor switch protein	✓
	cj0448c	*acfB*, accessory colonisation factor, MCP-type signal transduction protein	✓
	cj0526c	*fliE*, flagellar hook-basal body complex protein	✓
	cj0527c	*flgC*, flagellar basal body rod protein	✓
	cj0528c	*flgB*, flagellar basal body rod protein	✓
	cj0547	*flaG*, flagellar protein	✓
	cj0548	*fliD*, flagellar hook-associated protein	✓
	cj0549	*fliS*, flagellar protein	✓
	cj0670	*rpoN*, RNA polymerase sigma-54 factor	✓
	cj0687c	*flgH*, putative flagellar L-ring protein precursor	✓
	cj0697	*flgG2*, *flgF*, flagellar basal-body rod protein	✓
	cj0698	*flgG*, flgG, flagellar basal-body rod protein	✓
	cj0720	*flaC*, flagellin	✓

	cj0769c	*flgA*, putative flagellar protein	✓	
	cj0793	*flgS*, signal transduction histidine kinase	✓	
	cj0820c	*fliP*, flagellar biosynthesis protein	✓	
	cj0882c	*flhA*, flagellar biosynthesis protein	✓	
	cj0887c	*flaD*, *flgL*, flagellar hook-associated protein	✓	
	cj0923c	*cheR*, putative MCP protein methyltransferase	✓	
	cj0924c	*cheB*, putative MCP protein-glutamate methylesterase	✓	
	cj1024c	*flgR*, sigma-54 associated transcriptional activator	✓	
	cj1118c	*cheY*, chemotaxis regulatory protein	✓	
	cj1179c	*fliR*, flagellar biosynthesis protein	✓	
	cj1189c	*cetB*, bipartate energy taxis response protein	✓	
	cj1198	*luxS,* S-ribosylhomocysteine lyase	✓	
	cj1190c	*cetA*, bipartate energy taxis response protein	✓	
	cj1312	*pseG*, nucleotidase	✓	
	cj1313	*pseH*, N-acetyltransferase	✓	
	cj1331	*ptmB*, acylneuraminate cytidylyltransferase	✓	
	cj1332	*ptmA*, putative oxidoreductase	✓	
	cj1338c	*flaB*, flagellin		Missing in E00
	cj1339c	*flaA*, flagellin		Missing in E00
	cj1408	*fliL*, putative flagellar protein	✓	
	cj1462	*flgI*, flagellar P-ring protein	✓	
	cj1466	*flgK*, putative flagellar hook-associated protein	✓	
	cj1506c	*tlp1*, putative MCP-type signal transduction protein	✓	
	cj1565c	*pflA*, paralysed flagellum protein	✓	
	cj1675	*fliQ*, flagellar biosynthesis protein	✓	
	cj1729c	*flgE2*, flagellar hook subunit protein	✓	
adhesion	cj0289c	*peb3*, major antigenic peptide	✓	
	cj0921c	*peb1A*, aspartate/glutamate-binding ABC transporter protein	✓	
	cj0983	*jlpA*, surface-exposed adhesin		Missing in E00

	cj1259	*porA*, major outer membrane protein	✓		
	cj1279c	*flpA*, putative fibronectin domain-containing lipoprotein	✓		
	cj1351	*pldA*, phospholipase A	✓		
	cj1478c	*cadF*, outer membrane fibronectin-binding protein	✓		
	*CJJ81176_0067	*ggt*, gamma-glutamyltransferase		✓	
invasion	cj0914c	*ciaB*, invasion antigen B	✓		
	cj1647	*iamA*, putative ABC transport system ATP-binding protein	✓		
stress response	cj0012c	*rrc*, non-haem iron protein	✓		
	cj0020c	cytochrome C551 peroxidase	✓		
	cj0334	*ahpC*, alkyl hydroperoxide reductase	✓		
	cj0358	putative cytochrome C551 peroxidase	✓		
	cj0779	*tpx*, thiol peroxidase	✓		
	cj1260c	*dnaJ*, chaperone	✓		
	cj1272c	*spoT*, putative guanosine-3',5'-bis(diphosphate) 3'-pyrophosphohydrolase	✓		
	cj1385	*katA*, catalase	✓		
	*CJJ81176_1121	*csrA*, carbon storage regulator	✓		
iron acquisition	cj0178	putative TonB-dependent outer membrane receptor	✓		
	cj0755	*cfrA*, ferric enterobactin uptake receptor	✓		
	cj1355	*ceuE*, enterochelin uptake periplasmic binding protein	✓		
	cj1614	*chuA*, haemin uptake system outer membrane receptor	✓		
toxin	cj0077c	*cdtC*, cytolethal distending toxin C	✓		
	cj0078c	*cdtB*, cytolethal distending toxin B	✓		
	cj0079c	*cdtA*, cytolethal distending toxin A	✓		
type IV	**	*cmgB4*, putative type IV secretion system component, *virB4*			Present in B30, B31 and B44
secretion	**	*cmgB5*, putative type IV secretion system component, *virB5*			“
system	**	*cmgB6*, putative type IV secretion system component, *virB6*			“
(T4SS)	**	*cmgB7*, putative type IV secretion system component, *virB7*			“
	**	*cmgB8*, putative type IV secretion system component, *virB8*			“
	**	*cmgB9*, putative type IV secretion system component, *virB9*			“

	**	*cmgB10*, putative type IV secretion system component, *virB10*			“
	**	*cmgB11*, putative type IV secretion system component, *virB11*			“
	**	*cmgD4*, putative type IV secretion system component, *virD4*			“
	**pTet	pTet plasmid			“
	***tetO*	*tetO*, tetracyclin resistance gene	✓		
type VI	#	*tssA, VasJ*	✓		
secretion	#	*tssB, VipA*	✓		
system	#	*tssC, VipB*	✓		
(T6SS)	#	*tssD, Hcp*	✓		
	#	*tssE*	✓		
	#	*tssF, VasA*	✓		
	#	*tssG, VasB*	✓		
	#	*tagH, VasC*	✓		
	#	*tssI, VgrG*	✓		
	#	*tssJ, VasD*	✓		
	#	*tssK, VasE*	✓		
	#	*tssL*	✓		
	#	*tssM*	✓		
LOS class		A		✓	
		B			E38, E01, E03,
		C		✓	
		D			E79
		E			E00, B30, B31, B44
		F		✓	

Table S2. Results from comparative genomic analyses identifying genes unique for *C. jejuni* ST353CC isolates of different sequence types.

Annotation from BLAST of protein sequence	COG
**Unique genes for ST-9438 isolates**	
PseF, pseudaminic acid cytidylyltransferase, CMP-N-acetylneuraminic acid synthetase	**[M]** Cell wall/membrane/envelope biogenesis
restriction endonuclease subunit S, type I SS modification system specificity (S) subunit	**[V]** Defence mechanisms
class I SAM-dependent methyltransferase	**[H]** Coenzyme transport and metabolism
dihydroorotase, pyrimidine biosynthesis	**[F]** Nucleotide transport and metabolism
YeeE/YedE family protein, uncharacterized membrane protein	**[R]** General function prediction only
peptidase C39	**[R]** General function prediction only
HmcD domain-containing protein	**[C]** Energy production and conversion
relaxase/mobilisation nuclease domain-containing protein	**[X]** Mobilome: prophages, transposons
NTPase	**[F]** Nucleotide transport and metabolism
protein kinase family protein	**[T]** Signal transduction mechanisms
RadB, RecA-family ATPase	**[L]*** Replication, recombination and repair
resolvase, site-specific DNA recombinase	**[L]*** Replication, recombination and repair
VirB4, type IV secretion system conjugal transfer ATPase	**[U]** Intracellular trafficking, secretion, and vesicular transport
Rha family transcriptional regulator	**[X]** Mobilome: prophages, transposons
single-stranded DNA-binding protein	**[L]*** Replication, recombination and repair
VirB5, type IV secretion system P-type DNA transfer protein	**[U]** Intracellular trafficking, secretion, and vesicular transport
VirB6, type IV secretion system plasmid conjugal transfer protein	**[U]** Intracellular trafficking, secretion, and vesicular transport
VirB8, type IV secretion system DNA transporter protein	**[U]** Intracellular trafficking, secretion, and vesicular transport
VirB9, type IV secretion system P-type conjugative transfer protein	**[U]** Intracellular trafficking, secretion, and vesicular transport
VirB10, type IV secretion system protein	**[U]** Intracellular trafficking, secretion, and vesicular transport
VirB11, type IV secretion system P-type DNA transfer ATPase	**[U]** Intracellular trafficking, secretion, and vesicular transport
VirD4, type IV secretion system conjugative DNA transfer protein, TraG/TraD family ATPase	**[U]** Intracellular trafficking, secretion, and vesicular transport
type IV secretion system protein	**[U]** Intracellular trafficking, secretion, and vesicular transport
TrbM, conjugal transfer protein	**[U]** Intracellular trafficking, secretion, and vesicular transport
DNA topoisomerase III	**[L]*** Replication, recombination and repair

chaperone of the DnaK-DnaJ-GrpE chaperone system	**[E]** Protein fate, Protein folding and stabilization
repA, plasmid replication protein	**[L]*** Replication, recombination and repair
ParA family protein implicated in chromosome segregation	**[L]*** Replication, recombination and repair
type I restriction modification system DNA methylase	**[V]** Defense mechanisms
GNAT family N-acetyltransferase	**[G]** Carbohydrate transport and metabolism
**Unique genes for ST-9336, ST-9437 and ST-3515 isolates**	
TnpV, transposon-encoded protein	**[X]** Mobilome: prophages, transposons
MerR family of transcription regulatory protein	**[K]** Transcription
transposase	**[X]** Mobilome: prophages, transposons
class I SAM-dependent methyltransferase	**[H]** Coenzyme transport and metabolism
DNA methyltransferase	**[R]** General function prediction only
dihydroorotase	**[F]*** Nucleotide transport and metabolism

*Involved in information storage and processing

Figure S1. BRIG analysis of plasmids identified in *C. jejuni* ST353CC isolates B30, B31 and B44 (all ST-9438) compared to the pTet plasmid from *C. jejuni* 81-176. Outer ring shows consensus sequence with arrows indicating ORFs.

Figure S1

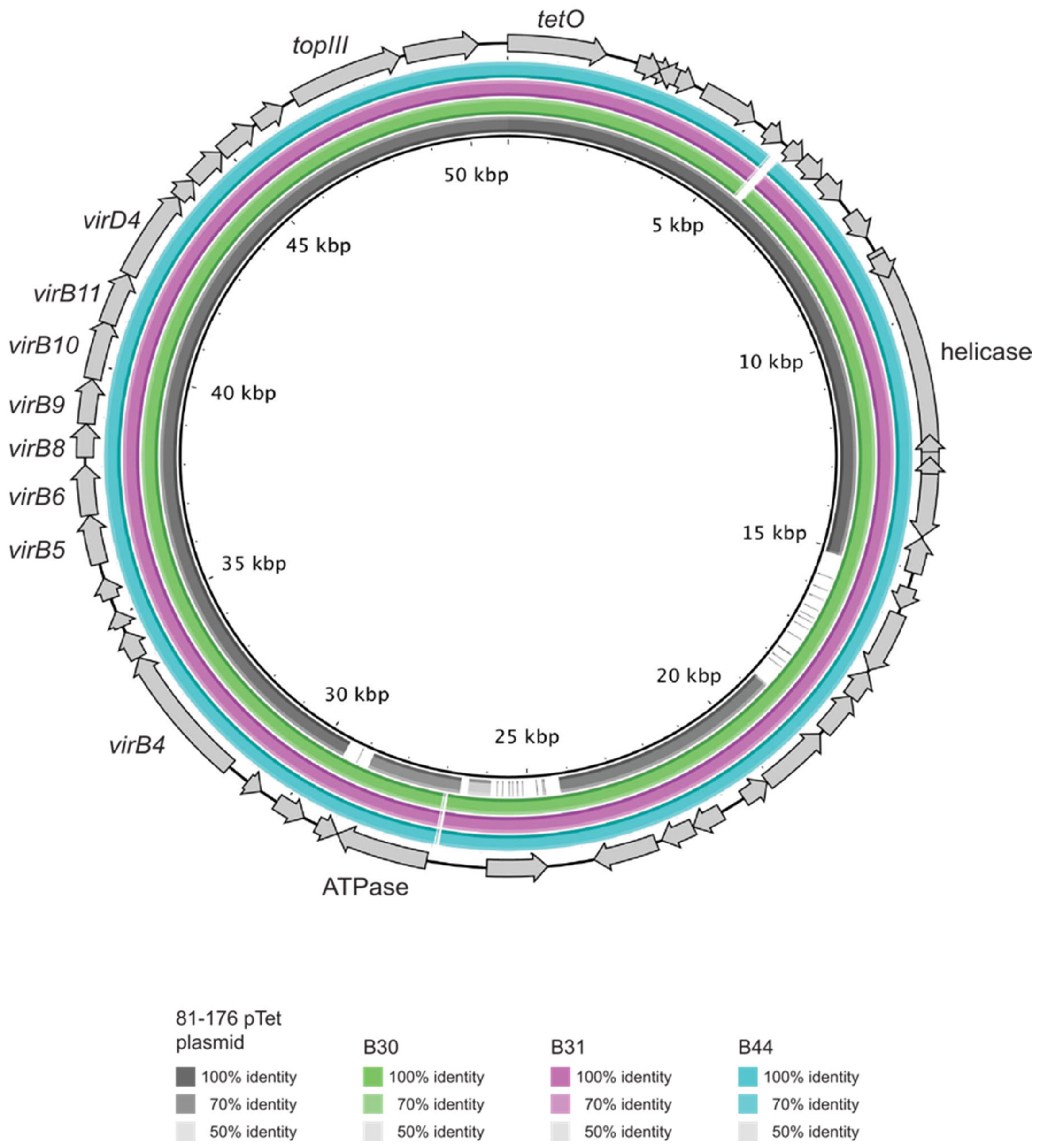

Figure S2. Chromosomal location of a 2862 nucleotide long fragment containing the tetO gene in non-ST-9438 isolates. The integration site contains several citrate transporters and is here theoretically demonstrated in the *C. jejuni* 11168 reference strain genome.

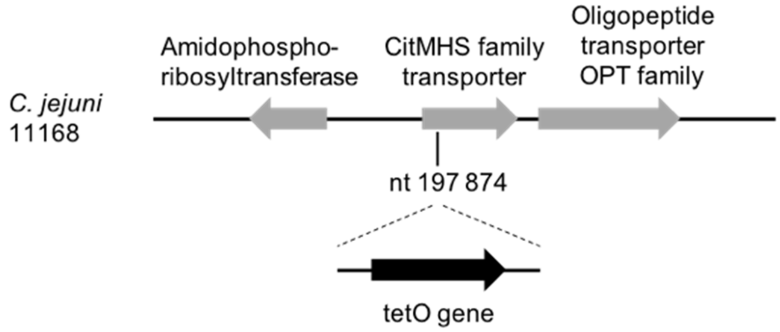

Supplement: Supplementary file 1 [file microorganisms-09-02540-s001.zip › microorganisms-1431505-supplementary.pdf]
